# When Aspirations Exceed Expectations: Quixotic Hope Increases Depression among Students

**DOI:** 10.1371/journal.pone.0135477

**Published:** 2015-09-09

**Authors:** Katharine H. Greenaway, Margaret Frye, Tegan Cruwys

**Affiliations:** 1 The University of Queensland, School of Psychology, Brisbane, Queensland, Australia; 2 Canadian Institute for Advanced Research: Social Interactions, Identity, and Well-being program, Toronto, Ontario, Canada; 3 Princeton University, Sociology Department, Princeton, New Jersey, United States of America; 4 Canadian Institute for Advanced Research: Successful Societies program, Toronto, Ontario, Canada; University of New South Wales, AUSTRALIA

## Abstract

A paradox exists in modern schooling: students are simultaneously more positive about the future and more depressed than ever. We suggest that these two phenomena may be linked. Two studies demonstrated that students are more likely to be depressed when educational aspirations exceed expectations. In Study 1 (*N* = 85) aspiring to a thesis grade higher than one expected predicted greater depression at the beginning and end of the academic year. In Study 2 (*N* = 2820) aspiring to a level of education (e.g., attending college) higher than one expected to achieve predicted greater depression cross-sectionally and five years later. In both cases the negative effects of aspiring high while expecting low persisted even after controlling for whether or not students achieved their educational aspirations. These findings highlight the danger of teaching students to aspire higher without also investing time and money to ensure that students can reasonably expect to achieve their educational goals.

## Introduction

In school, we teach kids to dream big, aim high, and shoot for the stars. This aspirational message is a mainstay of college graduation speeches [1] and is even espoused to our youngest students, from first grade onwards [2]. In line with these messages, educational aspirations have been steadily increasing—an effect termed “ambition inflation” [3]. Unfortunately, a number of barriers continue to prevent many students from *realizing* their aspirations. Moreover, this increasingly positive view of the future contrasts with the darker reality in schools that depression is on the rise. Roughly a third of college students report feeling depressed [4]. This figure is substantially higher than in the general population [5] and appears to be increasing [6].

We argue that what might appear as two disconnected news headlines are perhaps linked. Using a multi-method approach we find that educational aspirations that rise beyond realistic expectations are associated with greater depression now and in the future. Educational *expectations* are standards of educational attainment that individuals imagine they are likely to achieve, taking into account their current skills, dispositions, and opportunities. In contrast, educational *aspirations* are standards of attainment that individuals would choose to achieve under ideal conditions; they are not necessarily dependent on perceived probability of attainment [7,8,9]. Thus, aspirations and expectations can (and do) differ. For example, a student may aspire to ace an exam but hold more modest expectations for their performance. We propose that the more these judgements diverge—specifically, the more that aspirations outstrip expectations—the greater the likelihood of negative mental health outcomes in students.

### Negative Consequences of Aspiration

Across a variety of life domains, we are taught the value of positive thinking. This is a strategy espoused in self-help dogma [10,11] and enjoys particular popularity in rhetoric around health [12]. Yet, research now highlights downsides to “looking on the bright side”. For example, people with inflated self-esteem—an excessively positive view of the self—have been shown to react to failure and other ego threats with aggression and risk-taking, reflecting poor self-regulation [13,14]. Unregulated pursuit of happiness can, paradoxically, predict greater unhappiness [15,16]. Excessive positive thinking expressed in newspaper reports has even been associated with national economic downturn [17].

The potential dangers of dreaming big and aspiring high are also notable in academic domains, and appear to depend on the degree to which positive thinking is grounded in reality. For example, indulging in fantasies—idealized positive visions of the future—reduces effort to achieve important goals. Oettingen and Mayer [18] found that the more college students *fantasized* about doing well on an exam, the worse they performed. However, the better students *expected* to do on the exam, the better they performed. Fantasies are thought to provide similar emotional satisfaction as achieving a goal, inhibiting people from investing the effort needed to effectively pursue one’s goals [19,20].

Beyond self-regulation costs, positive aspirations can also have negative well-being consequences when ungrounded in reality. For example, wishful thinking is a relatively ineffectual coping strategy, predicting greater depression after stressful events [21]. Unrealistic idealization of one’s partner predicts declines in marital satisfaction over time [22]. Students who hope for academic outcomes better than they achieve show lower well-being than students who achieve their academic goals [23,24]. Indeed, Sweeny and Shepperd [25] found that optimism about future exam performance was associated with greater negative affect after the exam, regardless of how people actually performed.

Self-discrepancy theory [26] posits that people have an *actual self* (a person’s representation of their existing abilities and qualities) and an *ideal self* (a person’s representation of the abilities and qualities they aspire to possess). When these different self-representations contradict one another, people experience emotional discomfort. In particular, ideal-actual discrepancies are associated with feelings of dejection, disappointment, and depression [27]. This suggests that when students’ ideal selves (i.e., aspirations for educational attainment) appear out of reach based on what they can reasonably expect to achieve based on their actual circumstances (i.e., expectations for educational attainment), they are at risk of poor mental health outcomes. We apply this theorizing to understand how student populations can be simultaneously more depressed and more aspirational than ever. We argue that it is when educational aspirations exceed educational expectations that students are vulnerable to developing depression.

We term this discrepancy between aspiration and expectation *quixotic hope*. While hope is associated with greater feelings of efficacy [28], which are linked with expectations of success [29], hope also represents an aspiration that the future will be better than the present reality. Hope is experienced when people feel positively about a future event or outcome that is uncertain. Uncertainty is central to hope—hoping for an outcome does not guarantee that it will occur [30]. Hope thus straddles the divide between expectation and aspiration. It is therefore distinct from the related concepts of optimism and pessimism, which reflect an expectation that positive or negative outcomes will be forthcoming, with no reference to aspiration [31,32,33]. We describe the hope in our particular academic context as “quixotic” to reflect its idealized nature—the unrealistic outstripping of expectation by aspiration.

While previous research has investigated the gap between expectation and actual achievement [24,34,35,36], scholars have paid less attention to the mental health consequences of *aspiring* higher than one *expects*. This is despite the fact that it has been two decades since Hanson [7] first demonstrated that such discrepancies are widespread among youth. Recent cross-sectional evidence from US middle-school students suggests that discrepant aspirations and expectations predict diminished school bonding [37] and dissatisfaction with classroom experiences [38].

In the present research we explore the consequences of aspiring higher than one expects for the problem of depression in schools. We conceptualize the phenomenon of aspirations exceeding expectations as a discrepant mental state that may be associated with depressive styles of thinking, regardless of whether future performance meets one’s expectation. Although research shows that failure lowers well-being [23], we believe that the subjective feeling that one will not do as well as one hopes will be associated with depression independently of objective success or failure. Indeed, research shows that optimism about future exam performance is associated with greater post-exam negative affect even when controlling for performance outcome [25]. We therefore predicted that the negative impact of quixotic hope would occur independent of actual later achievement.

We explored the association between quixotic hope and depression cross-sectionally and longitudinally for two types of future educational outcomes: one that is short-term and specific (grade on a final-year thesis) and one that is long-term and diffuse (how far students aim to go in school). Specifically, we show the negative psychological consequences of quixotic hope among college students who aspire to perform well on their undergraduate thesis (Study 1), and among high school students who aspire to complete higher education (Study 2). In both cases, we demonstrate that experiencing quixotic hope—aspiring higher than one expects—predicts greater depression cross-sectionally and over time.

We hypothesized first that quixotic hope (reflected in a greater difference between academic aspirations and expectations) would be associated with greater depression at Time 1. We performed this analysis in both studies controlling for previous academic performance and academic expectations. We did this to show the importance of quixotic hope over and above simply expecting high or low grades—represented both as students’ self-reported expectations for future performance and what students could reasonably expect to achieve based on their academic track record.

We further hypothesized that quixotic hope at Time 1 would be associated with greater depression one year (Study 1) and five years (Study 2) in the future. In testing this hypothesis we controlled for actual educational attainment at Time 2 as well as academic expectations at Time 1. We did this to show that quixotic hope is associated with poorer mental health regardless of whether people meet the high aspirations that they hold for themselves.

## Study 1

Study 1 assessed the relationship between quixotic hope and depression among university students in their final year of a psychology degree. These students must complete an undergraduate thesis, which determines their eligibility for postgraduate study. At the beginning of the academic year, we assessed students’ levels of quixotic hope, operationalized as the difference between aspired and expected grade. We surveyed students again at the end of the academic year to explore whether this measure of quixotic hope predicted greater increases in depression over time while controlling for baseline levels of depression and actual performance. We predicted that when aspirations exceeded expectations, students would report greater depression cross-sectionally and over time.

## Method

### Ethics Statement

This study obtained ethical clearance from the Behavioural and Social Sciences Ethical Review Committee (BSSERC) at the University of Queensland. Before completing the questionnaire, participants were informed about the aims of the study, and written informed consent was obtained.

### Participants and Design

Participants were 85 fourth-year undergraduate psychology students (86% female, *M*_age_ = 23.13, *SD* = 4.93, age range 19 to 47) surveyed at the beginning of their Honours year. All students in the fourth-year cohort (approximately 120 students) were invited to participate. Students were given a gift voucher worth AU$20 for their participation in the second wave of the study, which was introduced as an opportunity for students to provide feedback about their final year experience. We sampled as many students as would complete the study and no observations were excluded from the results.

The study employed a longitudinal design. The Time 1 survey (*N* = 85) was completed three weeks into semester at the beginning of the academic year. Included in the Time 1 survey were measures of expected grade, aspired grade, previous academic performance, and depression.

Participants provided their email address at Time 1 to be contacted to participate at Time 2. The Time 2 survey (*N* = 38) was collected eight months after the Time 1 survey, a few weeks after participants discovered their final grade. Analyses revealed that attrition was not significantly related to variables of interest. Included in the Time 2 survey were measures of participants’ actual grade and depression. Both surveys included other measures unrelated to the current research question and not reported here.

### Time 1 Survey

#### Expected grade

Grade expectation was measured with the item “What result do you *expect* to get as your overall Honours mark?” ranging from 0 to 100.

#### Aspired grade

Grade aspiration was measured using one item (“What result do you *hope* to get as your overall Honours mark?”) on a scale ranging from 0 to 100.

#### Quixotic hope

A measure of quixotic hope was created by calculating the difference between aspired grade and expected grade. A positive score on this variable indicated that aspiration was higher than expectation, reflecting greater quixotic hope. A small proportion of our sample (*n* = 5) reported that their expectations exceeded their aspirations. We interpret this minority as “relaxed high performers” who comfortably expect high grades but acknowledge they would be happy with a lower grade than they expect. Because these responses capture an alternative mindset in which expectations exceed aspiration, we recoded these scores as 0 for the quixotic hopes measure and included a binary variable in the analyses that coded for participants whose expectations exceed their aspirations.

#### Previous grades

As a measure of previous academic performance, participants responded to the item “What grade did you get for third year statistics?” on a scale ranging from 0 to 100. This class was chosen because it is a required subject for all fourth year psychology students.

#### Depression

Depression was measured using the relevant subscale of the Depression, Anxiety and Stress Scale, short form (DASS-21; e.g., “I couldn’t seem to experience any positive feeling at all”; [39,40]). The measure is psychometrically robust, having excellent reliability and validity in both clinical and non-clinical samples. Participants reported how much the items had applied to them during the previous week on a scale ranging from 0 (*did not apply to me at all*) to 3 (*applied to me very much or most of the time)*. The items were summed and multiplied by 2 to form an index of depression as recommended by Henry and Crawford [39]. Higher scores indicate greater depression symptomatology.

### Time 2 Survey

#### Actual grade

Eight months later participants reported their final grade by responding to the item “What grade out of 100 did you get for your overall Honours mark?” on a scale ranging from 0 to 100.

#### Depression

Depression was measured in the same way as the Time 1 survey.

## Results

Table 1 contains descriptive statistics of the variables in the models. Tables 2 and 3 contain the bivariate correlations and results of the regression analyses, respectively. We use a significance threshold of *p* < .05 to evaluate the results. The correlation between expectations and previous grades was highly significant and positive (*r* = .89, *p* < .001) and significantly higher than the correlation between aspirations and previous grades (*r* = .54, *p* < .001), *z* = 5.24, *p* = .001. We found that on average, students expected a grade of 81.41 out of 100 for their final-year thesis, while they aspired for an average grade of 87.86. Students received an average grade of 78.00 for their thesis, suggesting that aspirations did outstrip expectations, which were on the whole relatively accurate.

**Table 1:** Means and standard deviations for measures included in the model (Study 1).

Variable	Possible range	Sample 1 Mean (SD)	Sample 2 Mean (SD)
*Psychological Well-being*			
Time 1 Depression	0 (normal) to 42 (extreme)	8.81 (9.20)	7.89 (7.95)
Time 2 Depression	0 (normal) to 42 (extreme)		8.63 (7.82)
*Aspirations and Expectations*			
Grade expectation	0 to 100	81.41 (6.75)	80.21 (6.89)
Grade aspiration	0 to 100	87.86 (5.92)	86.95 (6.17)
Quixotic hope (Aspiration > expectation)	0 (aspire = expect) to 30	6.60 (5.97)	
Expectations exceed aspirations (dummy)	0 (A > E) to 1 (E > A)	0.06 (0.24)	0.16 (0.37)
*Educational Performance*			
Previous year’s grade	0 to 100	81.69 (7.08)	80.32 (7.74)
Actual grade	0 to 100		78.00 (4.39)
*N*		85	38

**Table 2:** Bivariate correlations between the variables in the model (Study 1).

	1	2	3	4	5	6	7
1. Previous grades							
2. Expected grade	.89^***^						
3. Aspired grade	.54^***^	.53^***^					
4. Actual grade	.57^***^	.55^***^	.15				
5. T1 Depression	-.17	-.24^*^	.15	.01			
6. T2 Depression	.01	.08	.35^*^	-.09	.17		
7. E > A (dummy)	.21	.22^*^	-.13	-.04	.21	.17	
8. Quixotic hope	-.44^***^	-.58^***^	.38^***^	-.44^***^	.42^***^	.27^†^	-.28^**^

*Note*.

^***^ *p* < .001

^**^ *p* < .01

^*^ *p* < .05

^†^ *p* < .10

**Table 3:** Results of regression analyses for Study 1.

	Step 1	Step 2	Step 3
Wave 1 Depression			
Previous grades	-0.20 (-0.455, 0.059)	0.27 (-0.277, 0.818)	0.09 (-.435, 0.616)
Expected grade	-	-0.56 (-1.130, 0.019)	-0.11 (-0.717, 0.492)
E > A (dummy)	-	-	5.25 (-2.215, 12.721)
Quixotic hope (aspired-expected grade)	-	-	0.62^**^ (0.262, 0.980)
*R-Squared*	0.03	0.07	0.19
*F-test*	2.35	3.06^†^	4.82^**^
*N*	85	85	85
Wave 2 Depression			
T1 Depression	0.39^*^ (0.080, 0.707)	0.44^*^ (0.124, 0.748)	0.36^*^ (0.070, 0.656)
T1 Previous grades	0.08 (-0.256, 0.419)	-0.25 (-1.022, 0.515)	-0.49 (-1.227, 0.242)
T1 Expected grade	-	0.58 (-0.236, 1.394)	0.86^*^ (0.006, 1.713)
T2 Actual grade	-	-0.42 (-1.107, 0.258)	-0.07 (-0.737, 0.596)
E > A (dummy)	-	-	13.67 (-1.194, 28.539)
T1 Quixotic hope (aspired-expected grade)	-	-	0.52^*^ (0.077, 0.971)
*R-Squared*	0.16	0.23	0.41
*F-test*	3.26^*^	2.52^†^	3.54^*^
*N*	38	38	38

*Note*.

^**^ *p* < .01

^*^ *p* < .05

^†^ *p* < .10

The columns feature *b* weights with 95% confidence intervals in parentheses.

Quixotic hope was significantly *negatively* correlated with previous grades (*r* = -.44, *p* < .001), indicating that the more aspiration exceeds expectation, the less this hope appears to be based in reality. In addition, quixotic hope was positively correlated with depression at Time 1 (*r* = .42, *p* < .001) although at Time 2 the association was positive but not significant (*r* = .27, *p* = .084).

In the following regression analyses, we assessed the relationship between quixotic hope and depression cross-sectionally and longitudinally. In both sets of analyses, previous grades and expected grade was entered in initial steps followed by quixotic hope in another step to investigate the independent effect of quixotic hope. In the longitudinal analyses, Time 1 depression and attained thesis grade were added to the model to investigate *change* in depression over time as a function of quixotic hope, independent of how people actually performed. In both analyses we added a binary variable to indicate participants (*n* = 5) whose expectations exceeded their aspirations.

### Time 1 Depression

A hierarchical regression was conducted in which T1 depression was regressed onto previous grades in Step 1, expected grade in Step 2, and quixotic hope (the difference between aspired and expected grade) in Step 3. This analysis allows us to investigate the relationship between quixotic hope and depression when controlling the grade people expected to attain.

Previous grades did not predict depression in Step 1, *R* ^2^ = .03, *F*(1,83) = 2.35, *b* = -.20, ß = -.17, *p* = .129, 95% *CI*s for *b* = -0.455 to 0.059. Expected grades did not predict additional variance when entered in Step 2, *R* ^2^_∆_ = .04, *F*_∆_(1,82) = 3.06, *b* = -.55 ß = -.44, *p* = .058, 95% *CI*s for *b* = -1.130 to 0.019. Quixotic hope predicted depression when entered in Step 3, *R* ^2^_∆_ = .13, *F*_∆_(2,80) = 46.20, *b* = .62, ß = .44, *p* = .001, 95% *CI*s for *b* = 0.262 to 0.980, meaning that greater discrepancy between educational aspiration and expectation was associated with greater depression (see Table 3).

### Time 2 Depression

A hierarchical regression was conducted in which T2 depression was regressed onto T1 depression and previous grades in Step 1, expected grade and actual grade in Step 2 and quixotic hope (the difference between aspired and expected grade) in Step 3. This analysis allows us to investigate the relationship between quixotic hope and *change* in depression over time when controlling for expected performance, actual performance, and baseline depression.

Previous grades and T1 depression together predicted T2 depression in Step 1, *R* ^2^ = .16, *F*(2,35) = 3.26, *p* = .050, although only T1 depression independently accounted for variance, *b* = .39, ß = .40, *p* = .015, 95% *CI*s for *b* = 0.080 to 0.707. Actual grade and expected grade did not predict additional variance when entered in Step 2, *R* ^2^_∆_ = .08, *F*_∆_(2,33) = 1.65, *p* = .207. Quixotic hope predicted T2 depression when entered in Step 3, *R* ^2^_∆_ = .11, *F*_∆_(2,31) = 4.52, *b* = .52, ß = .44, *p* = .023, 95% *CI*s for *b* = 0.077 to 0.971, such that aspirations exceeding expectations at the start of the academic year was associated with an increase in depression eight months later.

## Study 2

Study 2 assessed quixotic hope in a nationally representative cohort of high school students in the United States reporting their aspirations and expectations for progression through the higher education system. We investigated the consequences of experiencing quixotic hope cross-sectionally and five years later. It was hypothesized that the more quixotic hope students experienced—the more educational aspirations exceeded expectations—the greater depression they would report now and in the future, controlling for expected and actual attainment.

## Method

### Ethics Statement

The survey data used for Study 2 were fully anonymized and cleared of identifying information before being released for general public access; thus no ethical clearance was required to use these data for the present study. The principal investigators for the National Survey of Youth and Religion (NSYR), Professors Christian Smith and Lisa Pearce, received ethical clearance from the University Review Board at the University of North Carolina. Prior to administering the surveys, interviewers obtained respondents’ verbal informed consent and provided respondents with information about the confidentiality of their answers and right to refuse to answer questions. To help protect the privacy of survey respondents, the NSYR obtained a Federal Certificate of Confidentiality from the National Institutes of Health. For more information about these and other measures taken to protect human subjects, visit the survey’s website at youthandreligion.nd.edu.

### Participants and Design

The data for Study 2 come from the National Survey of Youth and Religion (NSYR), a nationally representative telephone survey of 3,290 English and Spanish speaking teenagers aged 13–17, and their parents. The first wave of the NSYR was conducted from July 2002 to April 2003, and the third wave of the survey (used for the longitudinal analyses) was conducted between 2007 and 2008. The data have previously been used to study various aspects of adolescent social life, including socioeconomic disparities in educational aspirations and subsequent attainment [41], the impact of higher education on religious belief [42], substance use [43] and the relationship between cultural worldviews and social networks [44]. These data are ideal for this analysis because the survey contains detailed questions about aspirations and expectations regarding educational attainment, as well as questions that relate to psychological wellbeing and depression. Previous research comparing NSYR data with U.S. Census data on comparable households and with comparable adolescent surveys confirm that the NSYR provides a nationally representative sample without identifiable sampling and nonresponse biases of U.S. teenagers aged 13–17 living in households (for more information, see youthandreligion.org). Sampling weights are used to adjust for differential rates of sampling by census region, income groups, number of phone numbers in the household, and teenagers in the household.

The study employed a longitudinal design. The Time 1 sample comprised 2820 respondents who had valid responses for all variables included in the model (representing 86% of the total NSYR Wave 1 sample). Included in the Time 1 survey were measures of educational expectations, educational aspirations, previous academic performance, depression, and demographic controls.

The Time 2 sample comprised 1335 respondents who were successfully re-interviewed during Wave 3 of the NSYR, were at least 20 years of age and had valid responses for all variables included in the model (53% of the total NSYR Wave 3 sample). This age restriction was imposed to ensure that all respondents were at the age where they would have graduated from high school and started college if they remained “on track”, i.e. did not repeat any grades in school or take any years off. Nine hundred and ninety three respondents were excluded from the longitudinal analyses due to this age restriction. Two hundred and four respondents were excluded due to missing data on at least one variable used in the analyses. Included in the Time 2 survey were measures of attained level of education, depression, and demographics. The measures for Study 2 are outlined in Table 4.

**Table 4:** Means and standard deviations for measures included in Study 2.

Variable	Range	Time 1 Mean (se)	Time 2 Mean (se)
*Psychological Wellbeing*			
Time 1 Depression	1 (never) to 5 (always)	2.26 (0.87)	2.31 (0.82)
Time 2 Depression	1 (never) to 5 (always)		2.30 (0.74)
*Aspirations and Expectations*			
Educational Expectations	1 (high school) to 4 (graduate school)	2.88 (0.82)	2.96 (0.79)
Educational Aspirations	1 (high school) to 4 (graduate school)	3.05 (0.71)	3.09 (0.71)
Quixotic Hope (Aspirations–Expectations)	0 (aspiration = expectation) to 3 (aspire 3 levels higher)	0.22 (0.54)	0.16 (0.45)
Expectations exceed aspirations (dummy)	0 (A > E) to 1 (E > A)	3%	3%
*Sociodemographic Variables*			
Age	13 to 17	15.03 (1.38)	15.9 (0.87)
Female	Binary	50%	53%
Black	Binary	16%	14%
Hispanic	Binary	11%	8%
*Family Background*			
Annual Household Income	1 ($10,000) to 11 ($100,000 or above)	6.42 (3.22)	6.39 (2.94)
Parent Married or Cohabiting	Binary	74%	76%
At least one Parent Graduated from College	Binary	78%	81%
Family Stress in past year	1 (no stress) to 4 (a lot)	3.04 (0.89)	
*Educational Attitudes and Social Experiences*			
Grades Last Year	1 (Mostly Fs) to 10 (All As)	7.48 (1.55)	
“Mixed” Grades	Binary	6%	
Importance to Parent that Teen Graduates College	1 (Not important) to 5 (Extremely important)	4.50(0.73)	
In a Romantic Relationship	Binary	34%	
Time 2 Attended College	Binary	68%	
*N*		2822	1335

### Measures

#### Depression

The variable used to measure depression in the survey asked respondents to specify the frequency with which they experience negative emotions (“How often do you feel very sad and depressed?”) with responses ranging from 1 (*never*) to 5 (*always*). This measure was asked in the same way at Time 1 and Time 2.

#### Expected level of educational attainment

One item assessed educational expectations (“Given realistic limitations, how far in school do you think you actually *will* go?”; emphasis in the original survey instrument). We recoded this variable into four levels of academic credentials: 1 (*some high school or high school diploma*), 2 (*junior college or vocational school*), 3 (*four year college degree*), and 4 (*graduate school degree*).

#### Aspired level of educational attainment

One item measured educational aspirations (“Ideally, how far in school would you *like* to go?”). As with educational expectations, we recoded this variable into four levels of academic credentials: 1 (*some high school or high school diploma*), 2 (*junior college or vocational school*), 3 (*four year college degree*), and 4 (*graduate school degree*).

#### Quixotic hope

To construct a measure of quixotic hope, we calculated the difference between aspirations and expectations. This measure ranges from 0 (aspirations equal expectations) to 3 (aspire three levels higher than expect). A small proportion of our sample (*n* = 96) reported that they expected to go farther than they would ideally like to go. As in Study 1 we recoded these scores as 0 for the quixotic hopes measure and included a binary variable in the analyses identifying respondents whose expectations exceeded their aspirations.

#### Control Variables

Previous research suggests that educational aspirations and expectations are shaped by demographic characteristics, family background, educational attitudes and experiences [3]. Since these measures have also been linked to psychological wellbeing, their inclusion in our models is essential in order to examine whether expectations and quixotic hope may play an independent role in predicting psychological wellbeing. Demographic controls include age, gender, and binary indicators for black race and Hispanic ethnicity. Measures related to family background include parent-reported annual household income (ranging from less than $10,000 per year to more than $100,000 per year, in $10,000 categories) a binary variable indicating whether any parent graduated from college, a binary measure indicating whether the primary parent is either married or living with a partner, and parents’ responses to the question “In general, how much stress has your family been under in the past year?” ranging from 1 (*no stress*) to 4 (*a lot*).

Measures related to educational attitudes and experiences included the respondents’ estimate of the grades they usually received last year, ranging from 1 (*mostly ‘Fs’*) to 10 (*all ‘As’*). Six percent of respondents reported that their grades were “mixed” in a separate check box; these respondents were assigned the mean value of the grades measure and a binary indicator for this response was added to the models. To examine how parental pressure may influence both how respondents think about their own educational futures as well as their psychological wellbeing, parents were asked the question “How important or unimportant is it to you that your teen graduates from college?” with answers ranging from 1 (*not important at all*) to 5 (*extremely important*). Finally, a binary indicator assessed whether respondents were currently in a romantic or sexual relationship. The Time 2 survey measured educational attainment using a binary variable indicating whether respondents had ever attended a four-year college.

## Results

Table 4 contains descriptive statistics for the variables used in the models. Tables 5 and 6 contain bivariate correlations for the cross-sectional and longitudinal analyses, respectively. Tables 7 and 8 contain the regression results for the cross-sectional and longitudinal analyses, respectively. We use a significance threshold of *p* < .05 to evaluate the results. The correlation between educational expectations and previous grades was highly significant and positive (*r* = .34, *p* < .001) and significantly higher than the correlation between educational aspiration and previous grades (*r* = .27, *p* < .001), *z* = 2.90, *p* = .004. As in Study 1, evidence from this study suggests that the discrepancy between expectation and aspiration is more likely to be due to overly optimistic aspirations rather than pessimistic expectations. Specifically, among the 908 students who had attended college by Time 2, only 9 said that they expected to reach only a high school of education. Conversely, among the 433 students who had *never* attended college as of Time 2, only 57 (13%) said that they aspired to only a high school degree, while the remainder aspired higher than they attained.

**Table 5:** Bivariate correlations between the variables in the model (Study 2, Time 1).

	1	2	3	4	5	6	7	8	9	10	11	12	13	14	15	16	17
1. Depress.																	
2. Expect.	-0.14																
3. Aspir.	-0.08	0.70															
4. Quix hope	0.11	-0.56	0.14														
5. Exp > Asp	0.02	0.12	-0.23	-0.08													
6. Age	0.06	0.06	0.01	-0.08	-0.01												
7. Gender	0.13	0.09	0.09	-0.01	0.00	-0.01											
8. Black	-0.03	-0.04	-0.02	0.05	0.01	-0.01	0.02										
9. Hisp.	0.04	-0.06	-0.03	0.06	0.02	-0.04	0.03	-0.16									
10. Income	-0.08	0.25	0.20	-0.14	-0.03	0.05	-0.02	-0.21	-0.16								
11. Par mar.	-0.04	0.08	0.05	-0.06	-0.02	0.01	-0.01	-0.23	-0.04	0.44							
12.Par col.	-0.05	0.21	0.19	-0.08	-0.02	0.03	-0.03	-0.06	-0.18	0.40	0.18						
13. Fam stress	0.06	-0.03	-0.01	0.03	-0.01	0.01	0.00	-0.11	-0.06	-0.08	-0.06	0.03					
14. Grades	-0.10	0.34	0.27	-0.17	0.00	-0.01	0.16	-0.11	-0.05	0.22	0.13	0.13	-0.05				
15. Mix grades	0.01	-0.07	-0.05	0.04	-0.01	-0.07	-0.02	0.05	0.02	-0.05	-0.02	-0.04	-0.01	0.00			
16. Col. Impt.	-0.04	0.21	0.19	-0.05	0.02	-0.04	0.09	0.08	0.08	0.07	-0.01	0.06	-0.03	0.11	-0.01		
17. Dating	0.05	-0.02	-0.01	0.00	-0.02	0.24	0.06	0.05	-0.01	-0.02	-0.03	-0.03	-0.01	-0.06	-0.01	-0.04	

Note. The aspirations variable is not included in the regression models presented in Table 7; it is included here for reference.

**Table 6:** Bivariate correlations between the variables in the model (Study 2, Time 2).

	1	2	3	4	5	6	7	8	9	10	11	12	13	14
1. Dep.n T1														
2. Dep.n T2	0.33													
3. Expect. T1	-0.11	-0.09												
4. Aspir. T1	-0.07	-0.05	0.78											
5. Quix. Hopes T1	0.08	0.10	-0.49	0.10										
6. Exp. > Asp.	0.01	0.03	0.06	-0.25	-0.06									
7. Age	0.02	0.00	0.06	0.03	-0.06	-0.03								
8. Gender	0.16	0.12	0.10	0.08	-0.04	0.00	-0.05							
9. Black	-0.02	0.01	-0.07	-0.04	0.06	0.00	0.01	0.02						
10. Hisp.	0.05	0.01	-0.07	-0.02	0.08	0.01	-0.04	0.05	-0.12					
11. Income	-0.08	-0.11	0.28	0.22	-0.15	-0.02	0.03	-0.04	-0.21	-0.13				
12. Par. Mar.	-0.06	-0.03	0.21	0.18	-0.10	-0.01	0.00	-0.05	-0.09	-0.14	0.38			
13. Par. Col.	-0.05	-0.06	0.07	0.06	-0.03	0.00	-0.04	-0.03	-0.16	-0.04	0.45	0.15		
14. Attend. Col. T2	-0.08	-0.11	0.39	0.32	-0.18	-0.02	0.05	0.10	-0.07	-0.03	0.33	0.28	0.12	

Note. The aspirations variable is not included in the regression models presented in Table 8; it is included here for reference.

**Table 7:** Results of regression analyses for Study 2 Time 1 depression.

Variables	Step 1 Coeff. (CI)	Step 2 Coeff. (CI)	Step 3 Coeff. (CI)
*Aspirations & Expectations*			
Educational Expectations		-0.11***	-0.07*
		(-0.169, -0.051)	(-0.141, -0.005)
Quixotic Hope (Aspirations-Expectations)			0.11*
			(0.014, 0.207)
Exp. > Asp. (dummy)			0.148 (-0.061, 0.358)
*Sociodemographic Variables*			
Age	0.03^†^	0.03*	0.03*
	(-0.001, 0.055)	(0.003, 0.060)	(0.005, 0.062)
Female	0.24***	0.25***	0.24***
	(0.165, 0.319)	(0.170, 0.325)	(0.166, 0.321)
Black	-0.08	-0.08	-0.08
	(-0.195, 0.027)	(-0.191, 0.030)	(-0.194, 0.027)
Hispanic	0.09	0.09	0.08
	(-0.035, 0.214)	(-0.038, 0.208)	(-0.043, 0.200)
*Family Background*			
Annual Household Income	-0.02+	-0.01	-0.01
	(-0.032, 0.000)	(-0.027, 0.005)	(-0.027, 0.005)
Parent Married or Cohabiting	-0.00	-0.02	-0.01
	(-0.098, 0.093)	(-0.108, 0.081)	(-0.107, 0.082)
At least one Parent Graduated from College	-0.05	-0.02	-0.03
	(-0.150, 0.060	(-0.124, 0.083)	(-0.129, 0.077)
Family Stress	0.06**	0.06*	0.06*
	(0.014, 0.103)	(0.013, 0.083)	(0.013, 0.100)
*Education & Social Attitudes*			
Grades Last Year	-0.06***	-0.04***	-0.04**
	(-0.079, -0.030)	(-0.065, -0.012)	(-0.065, -0.012)
“Mixed” Grades	0.10	0.07	0.08
	(-0.083, 0.280)	(-0.101, 0.245)	(-0.097, 0.252)
Importance to Parent that Teen Graduates College	-0.03	-0.01	-0.01
	(-0.081, 0.026)	(-0.063, 0.045)	(-0.066, 0.041)
In a Romantic Relationship	0.04	0.04	0.04
	(-0.041, 0.123)	(-0.040, 0.123)	(-0.040, 0.123)
**Constant**	2.21***	2.22***	2.08***
	(1.663, 2.753)	(1.678, 2.768)	(1.514, 2.642)
*R-Squared*	0.05	0.06	0.06
*F-test*	8.31***	8.41***	7.77***
***N***	2,820	2,820	2,820

***p < .001

**p < .01

*p < .05

^†^ *p* < .1

Columns feature *b* weights with 95% CIs in parentheses.

**Table 8:** Results of regression analyses for Study 2 Time 2 depression.

Variables	Step 1 Coeff. (CI)	Step 2 Coeff. (CI)	Step 3 Coeff. (CI)
Time 1 Depression	0.28***	0.28***	0.27***
	(0.238, 0.331)	(0.230, 0.323)	(0.229, 0.322)
*Educational Attainment*			
Time 2 Attended College		-0.11*	-0.11*
		(-0.201, -0.018)	(-0.202, -0.019)
*Aspirations and Expectations*			
Educational Expectations		-0.03	-0.01
		(-0.086, 0.020)	(-0.065, 0.054)
Quixotic Hope (Aspirations-Expectations)			0.10*
			(0.002, 0.194)
Exp. > Asp. (dummy)			0.14 (-0.077, 0.363)
*Sociodemographic Variables*			
Age	0.00	0.00	0.01
	(-0.042, 0.043)	(-0.040, 0.045)	(-0.038, 0.047)
Female	0.11**	0.13**	0.13**
	(0.029, 0.182)	(0.049, 0.204)	(0.049, 0.204)
Black	-0.00	-0.00	-0.01
	(-0.113, 0.109)	(-0.115, 0.107)	(-0.120, 0.101)
Hispanic	-0.05	-0.04	-0.05
	(-0.186, 0.095)	(-0.183, 0.097)	(-0.191, 0.089)
*Family Background*			
Annual Household Income	-0.02**	-0.02^†^	-0.02^†^
	(-0.038, -0.007)	(-0.032, 0.001)	(-0.032, 0.001)
Parent Married or Cohabiting	-0.01	-0.02	-0.02
	(-0.111, 0.088)	(-0.119, 0.079)	(-0.120, 0.078)
At least one Parent Graduated from College	0.05	0.08	0.08
	(-0.056, 0.154)	(-0.025, 0.189)	(-0.027, 0.187)
**Constant**	1.70***	1.77***	1.62***
	(0.795, 2.609)	(0.866, 2.681)	(0.709, 2.540)
*R-Squared*	0.12	0.13	0.13
*F-test*	23.00***	19.46***	16.73***
*N*	1,335	1,335	1,335

*** *p* < .001

** *p* < .01

* *p* < .05

^†^ *p* < .1

Columns feature *b* weights with 95% CIs in parentheses.

Consistent with Study 1, quixotic hope was significantly *negatively* correlated with previous grades (*r* = -.17, *p* < .001), indicating that the more that aspiration exceeds expectation, the less this hope appears to be based in reality. In addition, quixotic hope was positively correlated with depression in both waves of the survey (*r*_T1_ = .11, *p* < .001; *r*_T2_ = .10, *p* < .001).

As in Study 1, we assessed the relationship between quixotic hope and depression both cross-sectionally and longitudinally. In both sets of analyses, demographic control variables were entered in Step 1 followed by educational expectations in Step 2 and quixotic hope (the positive difference between aspired and expected educational attainment) in Step 3. In the longitudinal analyses, Time 1 depression and four-year college attendance were added to the model to investigate *change* in depression over time as a function of quixotic hope, independent of the level of educational attainment that respondents had actually achieved. In both analyses we added a binary variable to indicate participants (*n* = 96) whose expectations exceeded their aspirations.

### Time 1 Depression

A hierarchical regression analysis was conducted in order to investigate the cross-sectional relationship between quixotic hope and depression when controlling for the demographic variables listed above. The demographic control variables predicted depression in Step 1, *R* ^2^ = .05, *F*(12,2807) = 8.31, *p* < .001 (see Table 7). In Step 2, educational expectations negatively predicted depression, *R* ^2^_∆_ = .01, *F*_∆_ (1,2806) = 13.21, *b* = -0.11, ß = -0.10, *p*<0.001, 95% *CIs* for *b* = -0.17 to -0.05.

As expected, quixotic hope predicted depression when entered in Step 3, *R* ^2^_∆_ = .003, *F*_∆_(2,2804) = 3.39, *b* = .11, ß = .07, *p* = .02, 95% *CI*s for *b* = 0.01 to 0.21, such that aspirations exceeding expectations was associated with greater depression.

### Time 2 Depression

A hierarchical regression was conducted in which Time 2 depression was regressed onto Time 1 depression, socio-demographic controls, and family background variables in Step 1, educational expectations and four-year college attendance in Step 2, and quixotic hope (the difference between aspired and expected educational attainment) in Step 3. This analysis allows us to investigate the relationship between quixotic hope and *change* in depression over time when controlling for expected attainment, actual attainment, and baseline depression.

Time 1 depression and the demographic controls predicted Time 2 depression in Step 1, *R* ^2^ = .12, *F*(8,1326) = 23.00, p < .001 (see Table 8). In Step 2, educational expectations and Time 2 college attendance predicted Time 2 depression, *R* ^2^_∆_ = .01 *F*_∆_(2,1324) = 4.77, *p* = .009, although only college attendance individually accounted for additional variance, *b* = -.11, ß = -.07, *p* = .019, 95% *CI*s for *b* = -0.20 to -0.02.

As expected, quixotic hope predicted Time 2 depression when entered in Step 3, *R* ^2^_∆_ = .004, *F*_∆_(2,1322) = 2.82, *b* = .10, ß = .06, *p* = .041, 95% *CI*s for *b* = 0.004 to 0.20, such that aspirations exceeding expectations at Time 1 was associated with an increase in depression five years later.

## General Discussion

A paradox exists in modern schooling: students are simultaneously more hopeful and more depressed than ever. Our findings suggest that these two phenomena are linked—when educational aspirations exceed expectations, students are more likely to present with depression. Two studies demonstrated that experiencing quixotic hope was associated with greater depression in the moment and over time among high school and college students. In Study 1, aspiring to a higher grade than one expected predicted depression at the beginning and end of the academic year. In Study 2, aspiring to a higher level of education than one expected predicted depression cross-sectionally and five years later. These effects were independent of whether people actually achieved their goal (i.e., received a high grade; attended college). Together, the results demonstrate that a mentality in which the goal a student *aspires* to achieve appears out of reach based on what they *expect* to achieve is associated with poorer mental health in the form of greater depression.

Previous research has shown that indulging in fantasies about future success undermines effort in the here-and-now [18]. Our findings suggest that this process may occur particularly when aspirations exceed expectations. Rather than treating these constructs as separate, we argue that it is the discrepancy between them—holding big dreams without expectation of success—that is particularly diagnostic of vulnerability.

It is possible that the combination of aspiring high while expecting low creates a mindset that reduces positive affect in the moment and over time. Freitas and Higgins [45] demonstrated that experiencing hope while in a vigilance mindset characterized by fear of failure was associated with less enjoyment of academic-related tasks. Hence, even if people achieve their goal, the seeds have already been sown for depressed affect. Other research suggests that motivational potential in the academic domain is realized particularly when people contemplate a situation in which they are likely to succeed (i.e., hold high expectations) and imagine a desirable outcome (i.e., hold high aspirations; [46]). This could explain why aspiring high while expecting low puts students at risk. This mindset has the potential to reduce self-regulatory effort *and* harm mental well-being, creating a spiral of failure and depression that is self-reinforcing.

Both studies relied on correlational data, and are therefore subject to the usual ambiguities regarding causality. It is possible that the experience of quixotic hope leads to depression as students believe that their hopes are unlikely to be realized—a possibility that our longitudinal analyses support. Of course, it is also possible that depressed students may hold lower expectations for themselves while holding high aspirations set by normative standards endorsed by peers, educators, and parents. It is not our intention to determine causality in this paper; simply the fact that the relationship exists between quixotic hope and depression poses a problem for students. In a time when students are encouraged to aspire higher [1,2,3], it is critical to understand the circumstances under which this message may be harmful to mental health.

Each study suffered from specific weaknesses that, when considered alone, limit our ability to draw firm conclusions. For example, Study 1 had a small sample which resulted in low power to detect effects, suffered from a high rate of attrition (over 50%), had limited demographic control variables and a relatively limited measure of previous academic performance (i.e., previous statistics grade). These limitations were addressed in Study 2, which had a large, nationally representative sample with ample demographic controls including a more representative measure of previous school grades. Study 2 had a main limitation in that the depression measure was not ideal, being only one item that was not clinically validated. This may explain the relatively modest levels of variance accounted for in this variable. However, obtaining the same results as Study 1, which included a well-validated depression measure, gives us confidence in the finding. The studies therefore complement one another in a variety of ways. Not only do the main strengths of each study compensate for the limitations of the other, the studies provide convergent evidence for our hypotheses using different time scales (one academic year in Study 1; five years in Study 2) and levels of specificity in educational goals (a specific piece of assessment in Study 1; level of educational attainment in Study 2). It is when considered *together* that the studies provide the most robust support for our theorizing.

To be clear, we are not suggesting that young people stop aiming high or dreaming big. Instead, we suggest that mere positive thinking is insufficient to overcome barriers to success, and thus can lead to disappointment. Our data reveal that students with quixotic hopes were more likely to come from low-income families, have parents who did not attend college, and to have previously experienced academic challenges. These students experience real structural barriers to educational attainment, as reflected in the discrepancy between their aspirations and expectations. Interventions should address these barriers to achievement, thus increasing the self-efficacy and capability of students to achieve their dreams.

Extensive research has shown that expectations rooted in feelings of self-efficacy encourage persistence and effort, and often yield academic success [47,18]. Even aspiration has been theorized to encourage feelings of agency and recognition of strategies to improve oneself [32,39]. Because it is the *discrepancy* between expectation and aspiration that makes students vulnerable, finding methods of reducing this gap—not by dampening aspirations but by boosting what students might reasonably expect to achieve—should have a positive effect on students’ mental health. It is possible that students can be “immunized” against developing aspirations that outstrip expectations in the same way that other educational interventions have been shown to improve mental health [48,49]. But to do so, practitioners and policy makers need to be aware that teaching students to dream big may have unintended negative consequences among those who are not yet capable of achieving their dreams.

## Supporting Information

S1 Dataset Data and syntax for Studies 1 and 2. (ZIP)
